# 10,16-Dichloro-6,20-dioxa-3,23-diaza­tetra­cyclo­[23.3.1.0^7,12^.0^14,19^]nona­cosa-1(29),7,9,11,14(19),15,17,25,27-nona­ene-4,22-dione methanol monosolvate

**DOI:** 10.1107/S1600536812007052

**Published:** 2012-02-24

**Authors:** Michaela Pojarová, Michal Dušek, Zdeňka Sedláková, Emanuel Makrlík

**Affiliations:** a Institute of Physics of the ASCR, Na Slovance 2, 182 21 Prague 8, Czech Republic; b Institue of Macromolecular Chemistry, ASCR v.v.i., Heyrovského nám. 2, 16202 Prague 6, Czech Republic; c Faculty of Environmental Sciences, Czech University of Life Sciences, Kamýcká 129, 165 21 Prague 6, Czech Republic

## Abstract

In the title compound, C_25_H_22_Cl_2_N_2_O_4_·CH_3_OH, the macrocyclic mol­ecule adopts a slightly distorted *C*_2_-symmetric conformation. The macrocyclic mol­ecules are linked *via* N—H⋯O hydrogen bonds between the amide groups into chains extending along the [010] direction. The methanol mol­ecules bridge these chains *via* N—H⋯O and O—H⋯O hydrogen bonds with the formation of a two-dimensional polymeric structure parallel to (001). The methanol mol­ecule is disordered over two positions with the occupancy ratio of 9:1. The disorder of the solvent molecule is caused by weak intermolecular C—H⋯Cl hydrogen bonding.

## Related literature

For application of macrocycles, see: Hayvali & Hayvali (2005 ▶); Kleinpeter *et al.* (1997 ▶); Jaiyu *et al.* (2007 ▶); Christensen *et al.* (1997 ▶); Alexander (1995 ▶). For the synthetic procedure, see: Ertul *et al.* (2009 ▶).
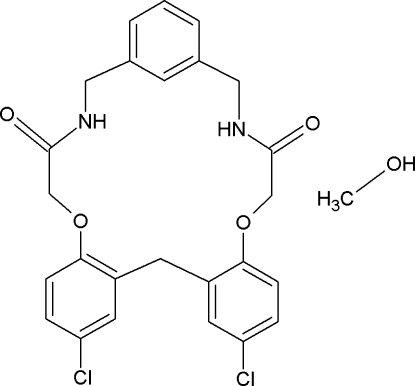


## Experimental

### 

#### Crystal data

C_25_H_22_Cl_2_N_2_O_4_·CH_4_O
*M* *_r_* = 517.39
Orthorhombic, 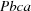

*a* = 21.9905 (3) Å
*b* = 8.1864 (1) Å
*c* = 26.6760 (3) Å
*V* = 4802.29 (10) Å^3^
*Z* = 8
Cu *K*α radiation
μ = 2.78 mm^−1^
*T* = 120 K
0.30 × 0.11 × 0.08 mm

#### Data collection

Oxford Diffraction Xcalibur A Gemini Ultra diffractometer
Absorption correction: multi-scan (*CrysAlis PRO*; Agilent, 2010 ▶) *T*_min_ = 0.826, *T*_max_ = 1.000
42959 measured reflections
4099 independent reflections
3364 reflections with *I* > 2σ(*I*)
*R*_int_ = 0.070

#### Refinement

*R*[*F* ^2^ > 2σ(*F* ^2^)] = 0.033
*wR*(*F* ^2^) = 0.082
*S* = 1.03
4099 reflections
326 parameters
4 restraints
H-atom parameters constrained
Δρ_max_ = 0.20 e Å^−3^
Δρ_min_ = −0.21 e Å^−3^

### 

Data collection: *CrysAlis PRO* (Agilent, 2010 ▶); cell refinement: *CrysAlis PRO*; data reduction: *CrysAlis PRO*; program(s) used to solve structure: *SHELXS97* (Sheldrick, 2008 ▶); program(s) used to refine structure: *SHELXL97* (Sheldrick, 2008 ▶); molecular graphics: *Mercury* (Macrae *et al.*, 2006 ▶) and *ORTEP-3* (Farrugia, 1997 ▶); software used to prepare material for publication: *publCIF* (Westrip, 2010 ▶).

## Supplementary Material

Crystal structure: contains datablock(s) I, global. DOI: 10.1107/S1600536812007052/gk2457sup1.cif

Structure factors: contains datablock(s) I. DOI: 10.1107/S1600536812007052/gk2457Isup2.hkl

Supplementary material file. DOI: 10.1107/S1600536812007052/gk2457Isup3.cml

Additional supplementary materials:  crystallographic information; 3D view; checkCIF report

## Figures and Tables

**Table 1:** Hydrogen-bond geometry (Å, °)

*D*—H⋯*A*	*D*—H	H⋯*A*	*D*⋯*A*	*D*—H⋯*A*
N1—H1N1⋯O5	0.98	2.31	3.039 (3)	130
N2—H1N2⋯O1^i^	0.93	2.20	2.860 (2)	128
O5—H1O5⋯O4^ii^	0.82	2.05	2.789 (3)	150
C26*A*—H26*F*⋯Cl2^iii^	0.96	2.74	3.616 (3)	149

Symmetry codes: (i) 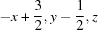; (ii) 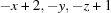; (iii) 
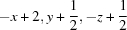
.

**Fig. 1.**
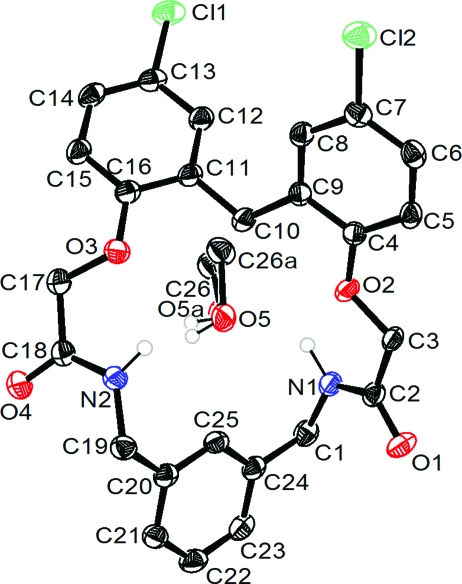
View of the asymmetric unit of the title compound with displacement ellipsoids shown at the 50% probability level. C-bound H atoms have been omitted for clarity.

**Fig. 2.**
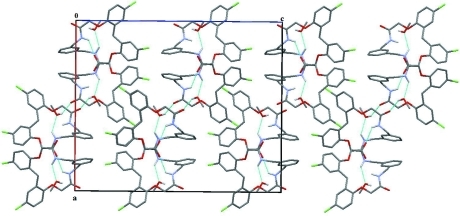
Projection along the b axis with highlighted hydrogen bonds between the molecules. Hydrogen atoms not involved in hydrgen bonding have been omitted.

## supplementary crystallographic information

### Comment

Polyazalactones together with polyoxalactones and polyethers are studied for their ability to act as multidentate ligands to complex various cations. Polyazalactones can incorporate transition metals into their cavities *via* an ion-dipole interaction (Hayvali & Hayvali, 2005; Kleinpeter *et al.*, 1997). They are studied for their role in bioprocesses, catalysis, material science, transport and separation (Jaiyu *et al.*, 2007; Christensen *et al.*, 1997; Alexander, 1995). In this paper, we report the crystal structure of a lactam ionophore (Fig.1). The macrocycle consists of three benzene rings, two of them substituted with chlorine atom in *para* position to O atom. The neighboring molecules are connected *via* hydrogen bonds between amide groups (Table 1). The crystal contains methanol molecule disordered over two positions with partial occupancies of 0.90 and 0.10. The hydroxyl group of the solvent forms hydrogen bond to the oxygen atom of the amide group (Table 1). The methyl group of the methanol in second position is also weakly bound to the chlorine atoms of neighboring molecule. This weak interaction competes with the stronger hydrogen bond to amide group and causes the solvent disorder.

### Experimental

All chemicals used were purchased from Fluka and used without further purification. The title compound was synthesized according to the method reported by Ertul *et al.* (2009). Single crystals were prepared by slow evaporation of methanol solution.

### Refinement

Positions of disordered groups were found from electron density maps. The disordered fragments were then placed in appropriate positions, and all distances between neighbouring atoms were restrained to 1.406 (20) Å. Site occupancies were refined for the different parts with the common displacement parameters for corresponding atoms in various fragments. At the end of the refinement, site occupancies were fixed at the values 0.9 and 0.1 and hydrogen atoms were placed in calculated positions. All hydrogen atoms of the macrocyclic molecule were found from electron density difference maps. H atoms attached to C atoms were placed in calculated positions. N—H distances were initially restrained to 1.00 Å with σ=0.02 and then fixed. The isotropic displacement parameters of H atoms were calculated as 1.2*U*_eq_ of the parent atom.

### Crystal data

**Table:**

C_25_H_22_Cl_2_N_2_O_4_·CH_4_O	*D*_x_ = 1.431 Mg m^−^^3^
*M**_r_* = 517.39	Melting point = 316–318 K
Orthorhombic, *P**b**c**a*	Cu *K*α radiation, λ = 1.5418 Å
Hall symbol: -P 2ac 2ab	Cell parameters from 6415 reflections
*a* = 21.9905 (3) Å	θ = 3.3–67.1°
*b* = 8.1864 (1) Å	µ = 2.78 mm^−^^1^
*c* = 26.6760 (3) Å	*T* = 120 K
*V* = 4802.29 (10) Å^3^	Prism, colourless
*Z* = 8	0.30 × 0.11 × 0.08 mm
*F*(000) = 2160	

### Data collection

**Table:**

Oxford Diffraction Xcalibur A Gemini Ultra diffractometer	4099 independent reflections
Radiation source: Enhance Ultra (Cu) X-ray Source	3364 reflections with *I* > 2σ(*I*)
Mirror monochromator	*R*_int_ = 0.070
Detector resolution: 10.3784 pixels mm^-1^	θ_max_ = 65.1°, θ_min_ = 3.3°
ω scan	*h* = −22→25
Absorption correction: multi-scan (*CrysAlis PRO*; Agilent, 2010)	*k* = −9→9
*T*_min_ = 0.826, *T*_max_ = 1.000	*l* = −29→31
42959 measured reflections	

### Refinement

**Table:**

Refinement on *F*^2^	Primary atom site location: structure-invariant direct methods
Least-squares matrix: full	Secondary atom site location: difference Fourier map
*R*[*F*^2^ > 2σ(*F*^2^)] = 0.033	Hydrogen site location: inferred from neighbouring sites
*wR*(*F*^2^) = 0.082	H-atom parameters constrained
*S* = 1.03	*w* = 1/[σ^2^(*F*_o_^2^) + (0.0358*P*)^2^ + 2.1383*P*] where *P* = (*F*_o_^2^ + 2*F*_c_^2^)/3
4099 reflections	(Δ/σ)_max_ = 0.002
326 parameters	Δρ_max_ = 0.20 e Å^−^^3^
4 restraints	Δρ_min_ = −0.21 e Å^−^^3^

### Special details

**Table:**

Geometry. All e.s.d.'s (except the e.s.d. in the dihedral angle between two l.s. planes) are estimated using the full covariance matrix. The cell e.s.d.'s are taken into account individually in the estimation of e.s.d.'s in distances, angles and torsion angles; correlations between e.s.d.'s in cell parameters are only used when they are defined by crystal symmetry. An approximate (isotropic) treatment of cell e.s.d.'s is used for estimating e.s.d.'s involving l.s. planes.
Refinement. Refinement of *F*^2^ against ALL reflections. The weighted *R*-factor *wR* and goodness of fit *S* are based on *F*^2^, conventional *R*-factors *R* are based on *F*, with *F* set to zero for negative *F*^2^. The threshold expression of *F*^2^ > σ(*F*^2^) is used only for calculating *R*-factors(gt) *etc*. and is not relevant to the choice of reflections for refinement. *R*-factors based on *F*^2^ are statistically about twice as large as those based on *F*, and *R*- factors based on ALL data will be even larger. Positions of disordered groups were found from electron density maps. The disordered fragments were then placed in appropriate positions, and all distances between neighbouring atoms were fixed. Site occupancies were refined for the different parts with the same thermal parameters for same atoms in various fragments. At the end of the refinement, site occupancies were fixed at values 0.90 and 0.10 and hydrogen atoms were placed into calculated positions. All hydrogen atoms could be found from maps of difference electron density, but those, attached to carbon atoms, were placed into calculated positions. The distance between N and H atoms were restrained to 1.00 Å with σ=0.02. The isotropic temperature parameters of hydrogen atoms were calculated as 1.2**U*_eq_ of the parent atom.

### Fractional atomic coordinates and isotropic or equivalent isotropic displacement parameters (Å^2^)

**Table:**

	*x*	*y*	*z*	*U*_iso_*/*U*_eq_	Occ. (<1)
Cl1	1.11069 (2)	0.02416 (7)	0.213524 (18)	0.03911 (14)	
Cl2	0.84287 (2)	−0.21013 (7)	0.147714 (18)	0.03856 (14)	
O1	0.70542 (6)	0.38705 (18)	0.40933 (5)	0.0367 (3)	
O2	0.80594 (5)	0.19883 (17)	0.32491 (5)	0.0305 (3)	
O3	0.96571 (6)	−0.16498 (18)	0.38954 (5)	0.0322 (3)	
O4	0.98144 (6)	−0.1787 (2)	0.52219 (5)	0.0406 (4)	
N1	0.80833 (7)	0.38887 (19)	0.40351 (6)	0.0277 (3)	
H1N2	0.8870	−0.1436	0.4391	0.033*	
N2	0.89913 (7)	−0.14110 (19)	0.47251 (5)	0.0262 (3)	
H1N1	0.8439	0.3425	0.3862	0.031*	
C1	0.81828 (9)	0.4614 (2)	0.45255 (7)	0.0315 (4)	
H1A	0.7853	0.5366	0.4597	0.038*	
H1B	0.8557	0.5239	0.4517	0.038*	
C2	0.75257 (8)	0.3497 (2)	0.38745 (7)	0.0266 (4)	
C3	0.74788 (8)	0.2590 (2)	0.33841 (7)	0.0265 (4)	
H3A	0.7195	0.1690	0.3417	0.032*	
H3B	0.7329	0.3318	0.3125	0.032*	
C4	0.81162 (8)	0.1097 (2)	0.28177 (6)	0.0240 (4)	
C5	0.76696 (8)	0.0977 (2)	0.24538 (7)	0.0267 (4)	
H5	0.7306	0.1547	0.2489	0.032*	
C6	0.77682 (8)	0.0000 (2)	0.20357 (7)	0.0289 (4)	
H6	0.7472	−0.0093	0.1788	0.035*	
C7	0.83113 (8)	−0.0828 (2)	0.19935 (7)	0.0276 (4)	
C8	0.87611 (8)	−0.0703 (2)	0.23554 (7)	0.0255 (4)	
H8	0.9124	−0.1273	0.2317	0.031*	
C9	0.86718 (8)	0.0266 (2)	0.27735 (7)	0.0242 (4)	
C10	0.91217 (8)	0.0476 (3)	0.31990 (7)	0.0338 (5)	
H10A	0.9167	0.1636	0.3263	0.041*	
H10B	0.8947	−0.0011	0.3498	0.041*	
C11	0.97467 (8)	−0.0239 (2)	0.31231 (7)	0.0283 (4)	
C12	1.00962 (8)	0.0216 (2)	0.27119 (7)	0.0296 (4)	
H12	0.9936	0.0927	0.2474	0.035*	
C13	1.06813 (8)	−0.0383 (2)	0.26540 (7)	0.0298 (4)	
C14	1.09288 (8)	−0.1440 (3)	0.29985 (7)	0.0317 (4)	
H14	1.1320	−0.1849	0.2953	0.038*	
C15	1.05893 (9)	−0.1890 (3)	0.34134 (7)	0.0313 (4)	
H15	1.0753	−0.2604	0.3649	0.038*	
C16	1.00041 (8)	−0.1277 (2)	0.34793 (7)	0.0275 (4)	
C17	0.99810 (9)	−0.1964 (3)	0.43499 (7)	0.0345 (5)	
H17A	1.0330	−0.1243	0.4370	0.041*	
H17B	1.0128	−0.3081	0.4348	0.041*	
C18	0.95804 (9)	−0.1705 (2)	0.48030 (7)	0.0291 (4)	
C19	0.85686 (9)	−0.1157 (2)	0.51386 (7)	0.0287 (4)	
H19A	0.8741	−0.1632	0.5440	0.034*	
H19B	0.8194	−0.1736	0.5066	0.034*	
C20	0.84194 (8)	0.0621 (2)	0.52404 (7)	0.0261 (4)	
C21	0.82821 (8)	0.1118 (3)	0.57256 (7)	0.0307 (4)	
H21	0.8297	0.0368	0.5987	0.037*	
C22	0.81235 (9)	0.2726 (3)	0.58206 (7)	0.0350 (5)	
H22	0.8037	0.3052	0.6147	0.042*	
C23	0.80922 (8)	0.3850 (3)	0.54350 (8)	0.0321 (4)	
H23	0.7985	0.4927	0.5503	0.039*	
C24	0.82213 (8)	0.3373 (2)	0.49450 (7)	0.0271 (4)	
C25	0.83856 (8)	0.1760 (2)	0.48543 (7)	0.0264 (4)	
H25	0.8475	0.1435	0.4528	0.032*	
O5	0.94522 (11)	0.3414 (4)	0.40875 (11)	0.0418 (7)	0.90
H1O5	0.9545	0.2931	0.4346	0.050*	0.90
C26	0.99892 (15)	0.3791 (5)	0.38117 (9)	0.0415 (7)	0.90
H26A	0.9904	0.4652	0.3578	0.050*	0.90
H26B	1.0123	0.2838	0.3633	0.050*	0.90
H26C	1.0303	0.4138	0.4039	0.050*	0.90
O5A	0.9587 (14)	0.374 (5)	0.4090 (12)	0.0418 (7)	0.10
H2O5	0.9634	0.2905	0.4254	0.050*	0.10
C26A	0.9961 (18)	0.367 (6)	0.3651 (11)	0.0415 (7)	0.10
H26D	0.9888	0.4625	0.3449	0.050*	0.10
H26E	0.9863	0.2711	0.3462	0.050*	0.10
H26F	1.0381	0.3642	0.3748	0.050*	0.10

### Atomic displacement parameters (Å^2^)

**Table:**

	*U*^11^	*U*^22^	*U*^33^	*U*^12^	*U*^13^	*U*^23^
Cl1	0.0270 (2)	0.0524 (3)	0.0380 (3)	−0.0081 (2)	0.00832 (19)	−0.0091 (2)
Cl2	0.0337 (3)	0.0480 (3)	0.0339 (3)	−0.0041 (2)	0.00026 (19)	−0.0116 (2)
O1	0.0250 (7)	0.0434 (9)	0.0417 (8)	0.0047 (6)	0.0079 (6)	−0.0067 (7)
O2	0.0183 (6)	0.0437 (8)	0.0294 (7)	0.0064 (6)	0.0010 (5)	−0.0059 (6)
O3	0.0221 (6)	0.0499 (9)	0.0245 (6)	0.0021 (6)	−0.0020 (5)	−0.0013 (6)
O4	0.0343 (8)	0.0595 (10)	0.0279 (7)	0.0096 (7)	−0.0066 (6)	0.0019 (7)
N1	0.0236 (8)	0.0284 (8)	0.0311 (8)	0.0029 (7)	0.0014 (6)	0.0015 (7)
N2	0.0254 (8)	0.0302 (9)	0.0230 (7)	0.0009 (7)	−0.0009 (6)	−0.0013 (6)
C1	0.0290 (10)	0.0283 (10)	0.0372 (11)	0.0017 (8)	−0.0014 (8)	−0.0044 (8)
C2	0.0242 (9)	0.0240 (10)	0.0315 (9)	0.0042 (8)	0.0035 (8)	0.0059 (8)
C3	0.0185 (8)	0.0298 (10)	0.0313 (9)	0.0057 (8)	0.0024 (7)	0.0050 (8)
C4	0.0216 (9)	0.0264 (9)	0.0241 (9)	−0.0010 (8)	0.0028 (7)	0.0051 (7)
C5	0.0184 (9)	0.0314 (10)	0.0302 (10)	0.0002 (8)	0.0002 (7)	0.0092 (8)
C6	0.0232 (9)	0.0359 (11)	0.0276 (10)	−0.0049 (8)	−0.0033 (7)	0.0064 (8)
C7	0.0261 (9)	0.0306 (10)	0.0260 (9)	−0.0053 (8)	0.0020 (7)	0.0013 (8)
C8	0.0208 (9)	0.0279 (10)	0.0277 (9)	−0.0002 (8)	0.0029 (7)	0.0043 (8)
C9	0.0194 (8)	0.0284 (10)	0.0247 (9)	−0.0012 (8)	0.0021 (7)	0.0058 (7)
C10	0.0218 (9)	0.0515 (13)	0.0280 (10)	0.0097 (9)	−0.0010 (8)	−0.0040 (9)
C11	0.0196 (9)	0.0398 (11)	0.0256 (9)	0.0026 (8)	−0.0024 (7)	−0.0082 (8)
C12	0.0233 (9)	0.0364 (11)	0.0290 (9)	0.0024 (8)	−0.0018 (8)	−0.0056 (8)
C13	0.0212 (9)	0.0383 (11)	0.0300 (10)	−0.0042 (8)	0.0019 (7)	−0.0109 (8)
C14	0.0186 (9)	0.0406 (12)	0.0359 (10)	0.0022 (8)	−0.0019 (8)	−0.0131 (9)
C15	0.0248 (10)	0.0374 (11)	0.0317 (10)	0.0055 (8)	−0.0048 (8)	−0.0076 (8)
C16	0.0207 (9)	0.0356 (11)	0.0262 (9)	−0.0002 (8)	−0.0017 (7)	−0.0074 (8)
C17	0.0294 (10)	0.0463 (13)	0.0278 (10)	0.0091 (9)	−0.0037 (8)	−0.0014 (9)
C18	0.0291 (10)	0.0313 (11)	0.0271 (10)	0.0028 (8)	−0.0027 (8)	0.0004 (8)
C19	0.0264 (9)	0.0326 (11)	0.0270 (9)	−0.0017 (8)	0.0027 (7)	0.0020 (8)
C20	0.0177 (8)	0.0335 (10)	0.0271 (9)	−0.0020 (8)	−0.0015 (7)	−0.0035 (8)
C21	0.0236 (9)	0.0434 (12)	0.0251 (9)	−0.0008 (9)	−0.0002 (7)	0.0004 (8)
C22	0.0286 (10)	0.0494 (13)	0.0271 (10)	0.0025 (9)	0.0009 (8)	−0.0109 (9)
C23	0.0235 (9)	0.0346 (11)	0.0383 (11)	0.0034 (9)	−0.0017 (8)	−0.0098 (9)
C24	0.0176 (9)	0.0334 (11)	0.0301 (10)	−0.0008 (8)	−0.0022 (7)	−0.0048 (8)
C25	0.0215 (9)	0.0334 (11)	0.0245 (9)	−0.0007 (8)	0.0011 (7)	−0.0047 (8)
O5	0.0243 (15)	0.0620 (19)	0.0390 (8)	0.0036 (10)	−0.0010 (10)	0.0116 (10)
C26	0.0367 (13)	0.0549 (17)	0.0330 (17)	−0.0057 (12)	−0.0024 (16)	0.0023 (19)
O5A	0.0243 (15)	0.0620 (19)	0.0390 (8)	0.0036 (10)	−0.0010 (10)	0.0116 (10)
C26A	0.0367 (13)	0.0549 (17)	0.0330 (17)	−0.0057 (12)	−0.0024 (16)	0.0023 (19)

### Geometric parameters (Å, º)

**Table:**

Cl1—C13	1.7470 (19)	C11—C16	1.395 (3)
Cl2—C7	1.7466 (19)	C12—C13	1.386 (3)
O1—C2	1.229 (2)	C12—H12	0.9300
O2—C4	1.368 (2)	C13—C14	1.375 (3)
O2—C3	1.415 (2)	C14—C15	1.385 (3)
O3—C16	1.381 (2)	C14—H14	0.9300
O3—C17	1.430 (2)	C15—C16	1.392 (3)
O4—C18	1.232 (2)	C15—H15	0.9300
N1—C2	1.338 (2)	C17—C18	1.511 (3)
N1—C1	1.453 (2)	C17—H17A	0.9700
N1—H1N1	0.9840	C17—H17B	0.9700
N2—C18	1.334 (2)	C19—C20	1.517 (3)
N2—C19	1.458 (2)	C19—H19A	0.9700
N2—H1N2	0.9295	C19—H19B	0.9700
C1—C24	1.514 (3)	C20—C21	1.390 (3)
C1—H1A	0.9700	C20—C25	1.391 (3)
C1—H1B	0.9700	C21—C22	1.385 (3)
C2—C3	1.507 (3)	C21—H21	0.9300
C3—H3A	0.9700	C22—C23	1.382 (3)
C3—H3B	0.9700	C22—H22	0.9300
C4—C5	1.384 (3)	C23—C24	1.394 (3)
C4—C9	1.404 (3)	C23—H23	0.9300
C5—C6	1.389 (3)	C24—C25	1.391 (3)
C5—H5	0.9300	C25—H25	0.9300
C6—C7	1.378 (3)	O5—C26	1.425 (3)
C6—H6	0.9300	O5—H1O5	0.8200
C7—C8	1.386 (3)	C26—H26A	0.9600
C8—C9	1.383 (3)	C26—H26B	0.9600
C8—H8	0.9300	C26—H26C	0.9600
C9—C10	1.516 (3)	O5A—C26A	1.430 (19)
C10—C11	1.508 (3)	O5A—H2O5	0.8200
C10—H10A	0.9700	C26A—H26D	0.9600
C10—H10B	0.9700	C26A—H26E	0.9600
C11—C12	1.390 (3)	C26A—H26F	0.9600
			
C4—O2—C3	118.82 (14)	C14—C13—C12	121.06 (18)
C16—O3—C17	116.49 (14)	C14—C13—Cl1	120.10 (14)
C2—N1—C1	121.63 (16)	C12—C13—Cl1	118.83 (16)
C2—N1—H1N1	119.0	C13—C14—C15	119.22 (17)
C1—N1—H1N1	117.4	C13—C14—H14	120.4
C18—N2—C19	121.82 (15)	C15—C14—H14	120.4
C18—N2—H1N2	115.1	C14—C15—C16	120.21 (19)
C19—N2—H1N2	123.0	C14—C15—H15	119.9
N1—C1—C24	113.56 (16)	C16—C15—H15	119.9
N1—C1—H1A	108.9	O3—C16—C15	122.18 (17)
C24—C1—H1A	108.9	O3—C16—C11	117.25 (16)
N1—C1—H1B	108.9	C15—C16—C11	120.58 (18)
C24—C1—H1B	108.9	O3—C17—C18	111.27 (15)
H1A—C1—H1B	107.7	O3—C17—H17A	109.4
O1—C2—N1	124.17 (18)	C18—C17—H17A	109.4
O1—C2—C3	118.49 (17)	O3—C17—H17B	109.4
N1—C2—C3	117.30 (16)	C18—C17—H17B	109.4
O2—C3—C2	109.29 (15)	H17A—C17—H17B	108.0
O2—C3—H3A	109.8	O4—C18—N2	123.83 (18)
C2—C3—H3A	109.8	O4—C18—C17	118.33 (17)
O2—C3—H3B	109.8	N2—C18—C17	117.84 (16)
C2—C3—H3B	109.8	N2—C19—C20	114.22 (15)
H3A—C3—H3B	108.3	N2—C19—H19A	108.7
O2—C4—C5	124.25 (16)	C20—C19—H19A	108.7
O2—C4—C9	114.12 (15)	N2—C19—H19B	108.7
C5—C4—C9	121.62 (17)	C20—C19—H19B	108.7
C4—C5—C6	119.53 (17)	H19A—C19—H19B	107.6
C4—C5—H5	120.2	C21—C20—C25	118.80 (18)
C6—C5—H5	120.2	C21—C20—C19	119.64 (17)
C7—C6—C5	118.96 (17)	C25—C20—C19	121.47 (16)
C7—C6—H6	120.5	C22—C21—C20	120.21 (19)
C5—C6—H6	120.5	C22—C21—H21	119.9
C6—C7—C8	121.69 (18)	C20—C21—H21	119.9
C6—C7—Cl2	119.10 (14)	C23—C22—C21	120.62 (18)
C8—C7—Cl2	119.19 (15)	C23—C22—H22	119.7
C9—C8—C7	120.19 (17)	C21—C22—H22	119.7
C9—C8—H8	119.9	C22—C23—C24	120.08 (19)
C7—C8—H8	119.9	C22—C23—H23	120.0
C8—C9—C4	118.01 (16)	C24—C23—H23	120.0
C8—C9—C10	125.23 (16)	C25—C24—C23	118.84 (18)
C4—C9—C10	116.75 (16)	C25—C24—C1	121.56 (17)
C11—C10—C9	116.76 (16)	C23—C24—C1	119.60 (18)
C11—C10—H10A	108.1	C24—C25—C20	121.43 (17)
C9—C10—H10A	108.1	C24—C25—H25	119.3
C11—C10—H10B	108.1	C20—C25—H25	119.3
C9—C10—H10B	108.1	C26—O5—H1O5	109.5
H10A—C10—H10B	107.3	C26A—O5A—H2O5	109.5
C12—C11—C16	118.45 (17)	O5A—C26A—H26D	109.5
C12—C11—C10	120.39 (18)	O5A—C26A—H26E	109.5
C16—C11—C10	121.00 (17)	H26D—C26A—H26E	109.5
C13—C12—C11	120.43 (19)	O5A—C26A—H26F	109.5
C13—C12—H12	119.8	H26D—C26A—H26F	109.5
C11—C12—H12	119.8	H26E—C26A—H26F	109.5

### Hydrogen-bond geometry (Å, º)

**Table:**

*D*—H···*A*	*D*—H	H···*A*	*D*···*A*	*D*—H···*A*
N1—H1*N*1···O5	0.98	2.31	3.039 (3)	130
N2—H1*N*2···O1^i^	0.93	2.20	2.860 (2)	128
O5—H1*O*5···O4^ii^	0.82	2.05	2.789 (3)	150
C26*A*—H26*F*···Cl2^iii^	0.96	2.74	3.616 (3)	149

Symmetry codes: (i) −*x*+3/2, *y*−1/2, *z*; (ii) −*x*+2, −*y*, −*z*+1; (iii) −*x*+2, *y*+1/2, −*z*+1/2.
